# Pulmonary hypertension and outcomes following left atrial appendage occlusion device placement for atrial fibrillation: A population‐based analysis

**DOI:** 10.1002/joa3.70071

**Published:** 2025-04-20

**Authors:** Nadhem Abdallah, Momen Alsayed

**Affiliations:** ^1^ Department of Internal Medicine Hennepin Healthcare Minneapolis Minnesota USA; ^2^ Banner University Medical Center University of Arizona Tucson Arizona USA

**Keywords:** atrial fibrillation, left atrial appendage occlusion, pulmonary hypertension

## Abstract

**Background:**

Pulmonary hypertension (PH) is linked to poor outcomes in cardiac procedures, but data on left atrial appendage occlusion device (LAAOD) placement are limited.

**Methods:**

Using the 2016–2020 Nationwide Readmission Database, we compared in‐hospital outcomes between AF patients with and without PH.

**Results:**

Among 48,692 AF‐LAAOD patients, 5.9% had PH. PH was associated with higher mortality, prolonged ventilation, AKI, vasopressor use, interatrial septum repair, LOS, and costs. No differences were found in the odds of readmissions, major bleeding events, vascular complications, stroke, or cardiac arrest.

**Conclusion:**

PH in AF‐LAAOD patients is associated with higher fatal and nonfatal adverse outcomes.

## Graphical abstract

Pulmonary hypertension in atrial fibrillation patients undergoing left atrial appendage occlusion is linked to higher mortality, complications, and healthcare costs, though readmission rates remain unchanged.

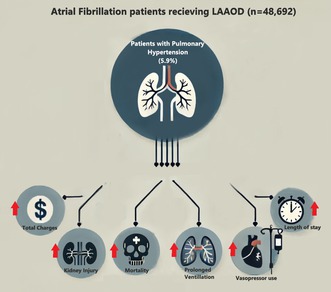

## 1 INTRODUCTION

Pulmonary hypertension (PH) is characterized by elevated pulmonary arterial pressure leading to right heart dysfunction. The relationship between PH and atrial fibrillation (AF) is well‐established in cardiovascular literature, with evidence showing PH contributes to both AF development and progression.^1^ The hemodynamic and structural cardiac changes associated with PH create an arrhythmogenic environment that complicates AF management. While previous research has documented poor outcomes in PH patients undergoing various cardiac procedures,^2^ analyses specifically examining PH's impact on left atrial occlusion device (LAAOD) placement outcomes remain limited. This knowledge gap is significant given the increasing use of LAAOD placement as a primary intervention for stroke prevention in patients with AF who are not candidates for anticoagulation. This study examines the association between PH and adverse outcomes in patients undergoing LAAOD placement by analyzing a large national dataset to identify risk factors and complications associated with PH in the context of LAAOD procedures.

## 2 MATERIALS, METHODS AND ETHICS STATEMENT

This study utilized the Nationwide Readmission Database (NRD) from January 1, 2016, to December 31, 2020. The NRD, developed by the Healthcare Cost and Utilization Project (HCUP) is a national inpatient database representing over 50% of the US population. The analysis incorporated NRD's hospital discharge weights, strata, and clusters to ensure national population representativeness and accurate statistical calculations, leveraging HCUP's weighting variables for precise estimation. Since the NRD contains de‐identified data that is publicly available, this study did not meet the definition of human subjects' research by the Hennepin Healthcare Institutional Review Board.

Adult patients (≥18 years) with a primary diagnosis of AF (ICD‐10‐CM code I48) who underwent LAAOD placement (ICD‐10‐PCS code 02L73DK) were identified and stratified based on the presence or absence of PH (ICD‐10‐CM code I27). The primary outcome was mortality, while secondary outcomes included prolonged ventilation (≥24 h), vascular complications requiring surgery, major bleeding events, percutaneous interatrial septum repair, vasopressor support, cardiac arrest, acute kidney injury (AKI), stroke, 90‐day readmissions, length of hospital stay (LOS), and total hospitalization charges (THC). To account for confounding variables, multivariate logistic and linear regression models were employed to adjust for age, gender, insurance status, median household income, and comorbidities (by the Deyo‐modified Charlson Comorbidity Index). All analyses were conducted using Stata 18.0, and statistical significance was defined as a *p*‐value <0.05.

## 3 RESULTS

**FIGURE 1:**
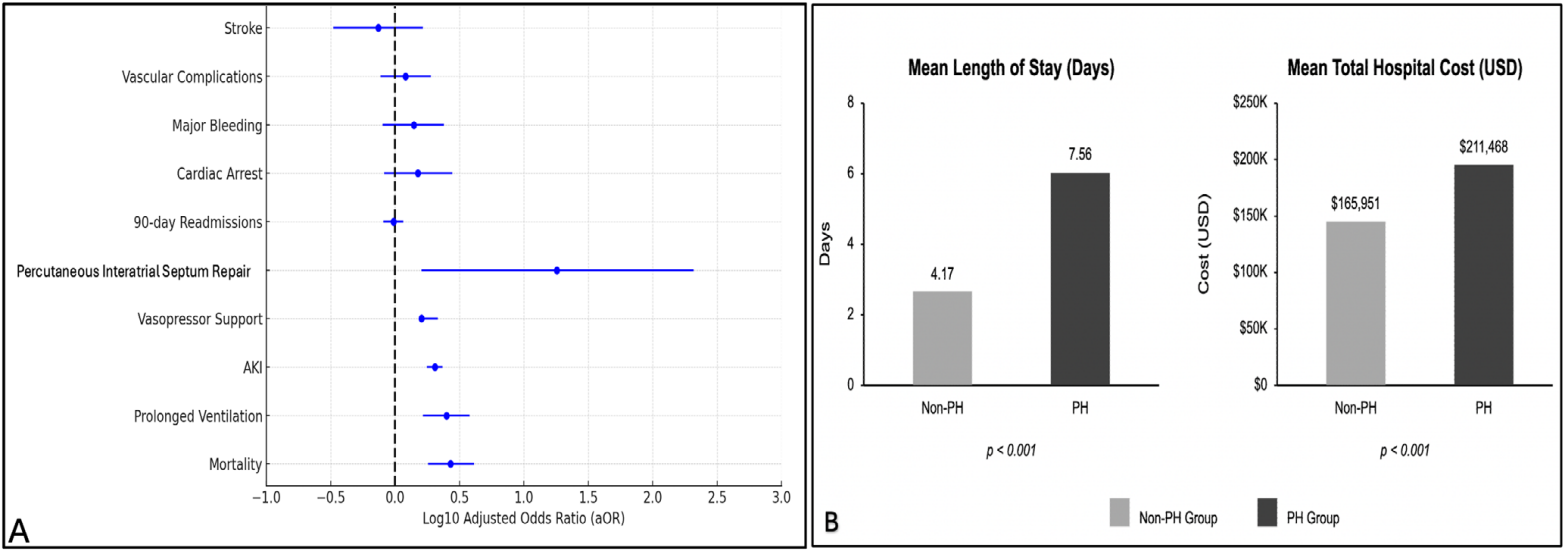
Clinical outcomes in patients undergoing LAAOD for atrial fibrillation. (A) Forest plot depicting adjusted odds ratios for categorical outcomes in patients with pulmonary hypertension versus those without. (B) Comparison of mean length of stay and total hospitalization costs between patients with and those without pulmonary hypertension.

Among 48,692 AF patients who underwent LAAOD placement, 5.9% (*n* = 2875) had PH. Patients with PH were older (71 vs. 67 years), more likely to be female (58% vs. 43%, *p* < 0.001), and had lower mean income quartiles (annual household income ≤$60,000, 55% vs. 49%, *p* < 0.001) compared to the non‐PH group. In adjusted analyses, PH was associated with higher odds of mortality (2.2% vs. 0.5%, adjusted odds ratio [aOR] 2.69, 95% confidence interval [CI] 1.80–4.10, *p* < 0.001). Additionally, PH was associated with increased odds of prolonged ventilation (2.6% vs. 0.8%, aOR 2.5, 95% CI 1.64–3.79, *p* < 0.001), AKI (30% vs. 12%, aOR 2.03, 95% CI 1.76–2.33, *p* < 0.001), vasopressor support (2.6% vs. 1.5%, aOR 1.6, 95% CI 1.60–2.15, *p* < 0.001), interatrial septum repair (0.1% vs. 0.01%, aOR 18, 95% CI 1.6–207, *p* = 0.02), longer LOS (7.56 vs. 4.17 days, *p* < 0.001), and higher THC ($211,468 vs. $165,951, *p* < 0.001) compared to no PH. No differences were observed in odds of 90‐day readmissions (13% vs. 10%, aOR 0.97, 95% CI 0.81–1.16, *p* = 0.74), cardiac arrest (1% vs. 0.5%, aOR 1.5, 95% CI 0.82–2.77, *p* = 0.19), major bleeding (1% vs. 0.6%, aOR 1.4, 95% CI 0.80–2.4, *p* = 0.23), vascular complications requiring surgery (1.5% vs. 1%, aOR 1.2, 95% CI 0.77–1.9, *p* = 0.36) or stroke (0.8% vs. 0.7%, aOR 0.74, 95% CI 0.33–1.64, *p* = 0.46) between both groups. Primary and secondary outcomes are presented in Figure 1.

## 4 DISCUSSION

Among AF patients undergoing LAAOD placement, PH was associated with higher odds of fatal and non‐fatal adverse events. The relationship between PH and AF is well‐documented in medical literature. A large‐scale analysis involving 152,385 patients who underwent AF ablation revealed that individuals with PH faced higher risks of adverse outcomes, including increased mortality and readmissions.^3^ Additional research has established that PH correlates with higher rates of atrial arrhythmia recurrence following cardiac interventions.^4^ This challenging relationship stems from complex pathophysiological mechanisms involving both structural and functional cardiac alterations. PH induces right atrial enlargement and elevated right heart pressures, creating an environment conducive to the development and persistence of atrial arrhythmias. Furthermore, the hemodynamic stress imposed by PH can compromise the effectiveness of LAAOD procedures, resulting in suboptimal therapeutic outcomes.^5^ Among AF patients undergoing LAAOD placement, PH was associated with higher odds of percutaneous interatrial septum closure, suggesting iatrogenic right‐to‐left shunting may occur following LAA occlusion in the setting of elevated pulmonary pressures. Further scholarship highlights that higher pulmonary artery pressures, especially persistent or newly developed PH, significantly increased mortality rates in cardiac procedures.^6^ These findings emphasize the need to stratify PH severity when assessing candidates for cardiovascular interventions, as greater PH severity can correlate with worse long‐term outcomes.

Moreover, among AF patients undergoing LAAOD placement, those with PH were more likely to be female and from lower‐income zip codes, suggesting potential gender and socioeconomic factors that may influence peri‐procedural outcomes. Existing literature supports these observations^7^ which emphasizes the importance of addressing social disparities in the management of AF‐PH patients undergoing LAAOD placement.

These findings should be interpreted considering the limitations of the NRD. Potential inaccuracies in ICD‐10 coding, lack of data on heart pressures, echocardiography, and PH etiology/severity, as well as the cause and timing of secondary diagnoses and study outcomes, along with the absence of long‐term follow‐up, may limit the analysis. Additionally, NRD does not include patient race. However, regular AHRQ quality checks help maintain data accuracy.

In conclusion, these findings emphasize that PH was associated with higher odds of fatal and non‐fatal adverse outcomes following LAAOD placement for AF. Further research is needed to validate these findings, accounting for PH subtypes, severity, and the role of social disparities in optimizing perioperative outcomes for this high‐risk population.

## FUNDING INFORMATION

None.

## CONFLICT OF INTEREST STATEMENT

Authors declare no conflict of interests for this article.

## ETHICS STATEMENT

Given that the data used in this study was derived from the Nationwide Readmissions Database, which is de‐identified and publicly accessible, this study did not require approval from any Institutional Review Board. Additionally, written informed consent from participants was not required in accordance with local and national guidelines.

## Data Availability

The data that support the findings of this study are not publicly available because of their containing information that could compromise the privacy of research participants. However, the data are available from the Healthcare Cost and Utilization Project (HCUP) Central Distributor upon reasonable request at https://www.hcup‐us.ahrq.gov/tech_assist/centdist.jsp. The underlying data used in this manuscript come from the Nationwide Readmissions Database (NRD), which is maintained by the Agency for Healthcare Research and Quality (AHRQ). Researchers can request access to the NRD through the HCUP Central Distributor by contacting them via email at hcup-requestdata@ahrq.gov.
